# Insulin-Like Growth Factor Receptor I (IGF-IR) and Vascular Endothelial Growth Factor Receptor 2 (VEGFR-2) Are Expressed on the Circulating Epithelial Tumor Cells of Breast Cancer Patients

**DOI:** 10.1371/journal.pone.0056836

**Published:** 2013-02-13

**Authors:** Monika Pizon, Dorothea Sonja Zimon, Ulrich Pachmann, Katharina Pachmann

**Affiliations:** 1 Transfusion Center Bayreuth, Bayreuth, Germany; 2 Clinic for Internal Medicine II, Oncology Research Laboratory, Friedrich Schiller University Jena, Jena, Germany; Johns Hopkins University, United States of America

## Abstract

**Background:**

Circulating epithelial tumor cell (CETC) analysis is a promising diagnostic field for estimating the risk for metastatic relapse and progression in patients with malignant disease. CETCs characterization can be used as a liquid biopsy for prognostic and predictive purposes in breast and other cancers. IGF-IR and VEGFR-2 play an important role in tumor growth and the progression of cancer disease. The purpose of the current study was therefore to investigate their expression on CETCs.

**Methods:**

CETCs were determined from the blood of 50 patients suffering from breast cancer. The number of vital CETCs and the expression of IGF-IR and VEGFR-2 were investigated using the maintrac*®* method.

**Results:**

IGF-IR and VEGFR-2 expression on the surface of CETCs were detected in 84% of patients. A statistically high correlation was found between IGF-IR and VEGFR-2 (r = 0.745 and p<0.001) on the CETCs. The co-expression of both receptors was confirmed in some experiments and ranged between 70% and 100%. Statistically significant correlations were observed between the number of CETCs and IGF-IR (r = 0.315 and p<0.05) and VEGFR-2 (r = 0.310 and p<0.05) expression. The presence of CETCs and the level of IGF-IR and VEGFR-2 expression were not associated with tumor stage, hormone receptor status or nodal/distant metastasis.

**Summary:**

In this study, a parallel and co-expression of IGF-IR and VEGFR-2 was examined on the surface of CETCs in breast cancer patients for the first time. Characterization of CETCs may be a promising approach for the rational design of targeted anticancer therapies.

## Introduction

Breast cancer is one of the most common cancers among women in the Western world. Despite improvements in early diagnosis and clinical management, breast cancer kills more than 520,000 people worldwide each year. Most breast cancer deaths are due to recurrent and metastatic disease [1]. The hypothesis that circulating tumor cells (CTCs) are associated with the development of metastasis was first proposed in 1869 by Thomas Ashworth [2]. Tumor cells are shed by both primary and metastatic cancers into the blood and are thought to mediate the hematogenous spread of cancer to distant sites, including the bones, lungs, brain and liver [3]. In many studies, the detection of tumor cells in blood in early and metastatic diseases has been shown to correlate with an unfavorable clinical outcome [4]. The detection of circulating tumor cells seems to predict progress in metastatic breast cancer [5]. Using a nondissipative approach (maintrac*®*), the enumeration of circulating epithelial tumor cells (CETCs) in patients with many types of cancer can be expected to contribute to monitoring the behavior of CETCs in patients with primary breast cancer during therapy [6], and directly reflects the patient’s response or lack of response to therapy. Moreover, enumeration and further characterization of CETCs can be used as liquid biopsy for repeated follow-up examinations in a variety of human cancers [7]. The phenotypical variety of breast cancer cells in primary tumors as well as in CETCs has been shown for well-known prognostic factors and could provide a very important tool for the development of new therapeutic strategies [8]. Two receptors, IGF-IR and VEGFR-2, have been shown to play an important role in the growth of the primary tumor and metastasis formation. IGF-IR belongs to the family of transmembrane receptor tyrosine kinases and is expressed on the cell surface of most tissues. Physiologically, this receptor and its ligands play a key role in the regulation of growth and metabolism. It has recently been demonstrated that IGF-IR is also a key player in cancer development and progression [9]. VEGFR-2 is also a transmembrane receptor that has an important role in endothelial cell development [10]. The majority of VEGFR-2 actions are related to angiogenesis, which is a critical event in tumor progression and metastasis [11]. In addition to its function in angiogenesis, VEGF signaling has been implicated in the ability of breast cancer to proliferate, evade apoptosis and migrate. The VEGFR-2 receptors are widely expressed in breast cancer and also in other tumors including lung, colon, uterus and ovarian cancers [12]. In the present study, we have used the maintrac*®* approach [13], to determine the expression of IGF-IR and VEGFR-2 on CETCs in the peripheral blood of patients with breast cancer.

## Methods

**Figure 1:**
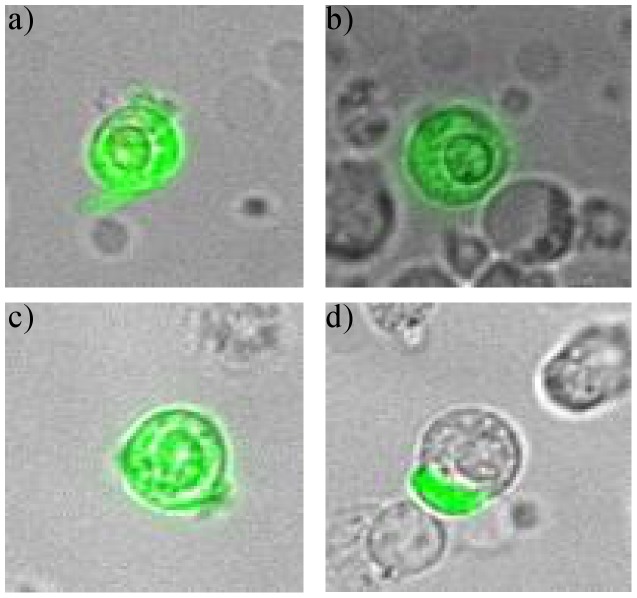
Immunostaining of CETCs with anti-EpCAM-FITC- and anti-IgG1-PE-antibody. There is no unspecific staining with anti-IgG1 (mouse)-PE- antibody. a) Labeling of the whole cell membrane with an additional cap; b),c) differently intense labeling of the cell membrane because EpCAM-fluorescence varies strongly between the individual cells; d) only exclusively surface located cap is stained.

**Figure 2:**
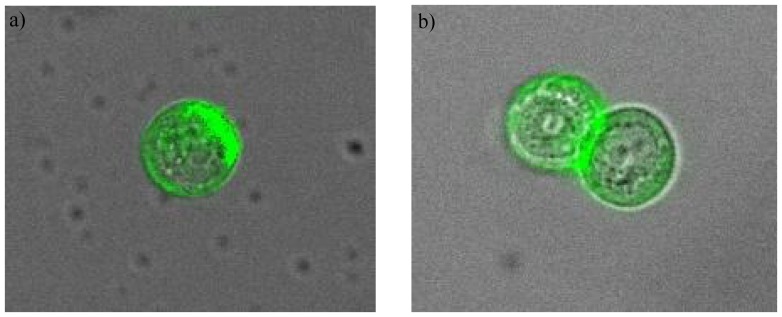
Immunostaining of MCF-7 cell line cells with anti-EpCAM-FITC- together with a) anti-IGF-IR-PE- or b) anti-VEGFR-2-PE-antibodies. MCF-7 cell line also expresses green EpCAM staining heterogeniously, similar to CETCs (positive control) and doesn’t show labeling for IGF-IR or VEGFR-2 (negative control).

50 patients with histologically confirmed breast cancer were enrolled in the study. Blood samples (2–7 ml) were drawn into normal blood count tubes with ethylenediaminetetraacetic acid (EDTA) as an anticoagulant and processed within 48 hours of collection. The maintrac*®* approach was used to enumerate CETCs, as reported previously [13] and to further characterize these cells. In brief, 1 ml blood was subjected to red blood cell lysis using 15 ml of erythrocyte lysis solution (Qiagen, Hilden, Germany) for 15 min in the cold, spun down at 700 g and re-diluted in 500 ml of PBS-EDTA. 5 µl of fluorescein-isothiocyanate (FITC)-conjugated mouse anti-human epithelial antibody (EpCAM) (Miltenyi Biotec GmbH, Germany) were added and incubated for 15 min in cold. The samples were subsequently diluted with 430 µl PBS-EDTA and then stored overnight at 4°C. A defined volume of the cell suspension and propidium iodide (PI) (Sigma-Aldrich, USA) was transferred to wells of ELISA plates (Greiner Bio-one, USA). Analysis of red and green fluorescence of the cells was performed using a Laser Scanning Cytometer*®* (Compucyte Corporation, Cambridge, USA), enabling relocation of cells for visual examination of vital epithelial cells. Vital CETCs were defined as EpCAM-positive cells, lacking in nuclear PI staining and with intact morphology (Figure 1), and only these cells were counted. We used isotype control as negative control to measure the level of non-specific background signal. Therefore samples were incubated with FITC-conjugated anti-EpCAM together with anti-IgG1 (mouse)-PE- antibody (Beckman Coulter) (Figure 1). Fluorospheres (Flow-Check 770, Beckman Coulter) were used for daily verification of LSC*®* optical components and detectors, which are required to ensure consistent analysis of samples. MCF-7 cell line was used as positive control for EpCAM expression and also as negative control for IGF-IR and VEGFR-2 staining of the CETCs (Figure 2).

**Figure 3:**
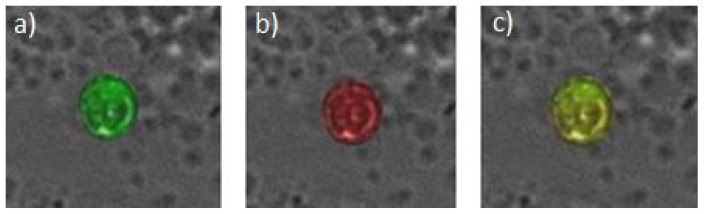
Immunostaining of CETC with anti-EpCAM-FITC and anti-IGF-IR-PE antibodies. a) Typical epithelial antigen-positive cell with green fluorescence. b, c) positive EpCAM CETC, which also has a red surface staining for IGF-IR.

**Figure 4:**
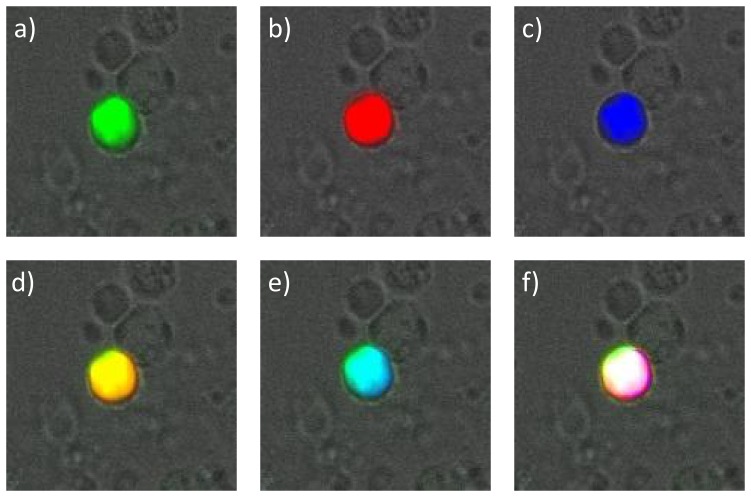
Fluorescence co-localization of EpCAM, IGF-IR and VEGFR-2 expression on the CETCs. a) Typical CETC with EpCAM green staining, b) the same CETC with IGF-IR red staining, c) CETC with VEGFR-2 blue staining d) merge of EpCAM and IGF-IR expression, e) merging of EpCAM and VEGFR-2 expression and f) merging of EpCAM, IGF-IR and VEGFR-2 expression on the same CETC.

The analyses of IGF-IR and VEGFR-2 expression on the CETCs were performed with an extended maintrac*®* approach from the same 50 patients. The samples were prepared as described above until the step of adding 500 µl PBS-EDTA. The samples were then divided in two. To each of the samples 5 µl phycoerythrin (PE)-labeled monoclonal mouse-antibody anti-VEGFR-2 (BD Bioscience, USA) or 5 µl of (PE)-labeled monoclonal mouse antibody anti-IGF-I receptor (BD Bioscience, USA) were added. Additionally all samples were incubated with 5 µl of (FITC)-conjugated mouse anti-human epithelial antibody (EpCAM) for 15 min in cold and then readjusted to 500 µl with PBS-EDTA and stored overnight at 4°C. For the measurement a defined volume of the cell suspension was transferred to wells of ELISA plates and measured with the Laser Scanning Cytometer (LSC). Subsequently, cells were visually inspected looking for a green and red surface staining, but also a well-preserved nucleus (Figure 3). In some experiments the co-expression of both receptors was evaluated using triple staining with FITC-conjugated anti-EpCAM antibody, PE-labeled anti-IGF-IR antibody and Pacific Blue conjugated mouse anti-VEGFR-2 antibody (BioLegend, USA). The samples then were measured with the LSC and cells were examined looking for green, red and blue fluorescence (Figure 4). Finally, the results for IGF-IR and VEGFR-2 were calculated as percentage of CETCs.

## Results

Among the 50 patients there were 28 (56%) patients with T1, 9 with T2 (18%), and 13 with T3/T4 (26%) tumor size. The age of patients ranged from 32 to 78 years (median 60). The primary tumors were histologically positive for ER in 76.0% of patients, positive for PR in 68.0% of patients, and positive for HER-2/neu in 28.0% of patients. The median of CETC was 35 per 100 µl of cell suspension (range 3–254). No statistically significant differences in CETC numbers were observed according to tumor size, lymph node status, presence of metastasis or clinical pathology (p>0.05) (Table 1).

**Table 1:** Baseline patient characteristics in relation to CETCs and IGF-IR/VEGFR-2 expression.

Primary tumor characteristics	Number of patients with CETCs (%)	p-value	Number of patients with positive IGF-IR (%) on CETCs	p-value	Number of patients with positive VEGFR-2 (%) on CETCs	p-value
Tumor size		0.375		0.729		0.158
						
T1	28 (56)		24/28 (85.7)		25/28 (89.3)	
T2	9 (18)		7/9 (77.7)		7/9 (77.7)	
T3/4	13 (26)		11/13 (84.6)		10/13 (76.9)	
Lymph node status		0.298		0.853		0.815
						
Positive						
Negative	27 (54)		20/27 (74)		20/27 (74)	
	23 (46)		22/23 (95.6)		22/23 (95.6)	
Metastasis		0.780		0.598		0.879
						
Positive	10 (20)		7/10 (70)		6/10 (60)	
Negative	38 (76)		23/38 (60.5)		24/38 (63.1)	
n.a.	2		2		2	
ER status		0.278		0.545		0.184
						
Positive	38 (76)		34/38 (89.5)		34/38 (89.5)	
Negative	10 (20)		9/10 (90)		9/10 (90)	
n.a.	2		2		2	
PR status		0.514		0.089		0.938
						
Positive	34 (68)		31/34 (91.2)		31/34 (91.2)	
Negative	14 (28)		12/14 (85.7)		12/14 (85.7)	
n.a.	2		2		2	
Her-2/neu expression		0.684		0.826		0.618
						
Positive (2+/3+)						
Negative (0/1+)	14 (28)		11/14 (78.6)		11/14 (78.6)	
n.a.	34 (68)		32/34 (94.1)		32/34 (94.1)	
	2		2		2	

Data presented as n (%). CETCs-circulating epithelial tumor cells; ER- estrogen receptor; PR- progesterone receptor; Her-2/neu- human epidermal growth factor receptor; IGF-IR – insulin-like growth factor I receptor; VEGFR-2 - vascular endothelial growth factor receptor-2

**Figure 5:**
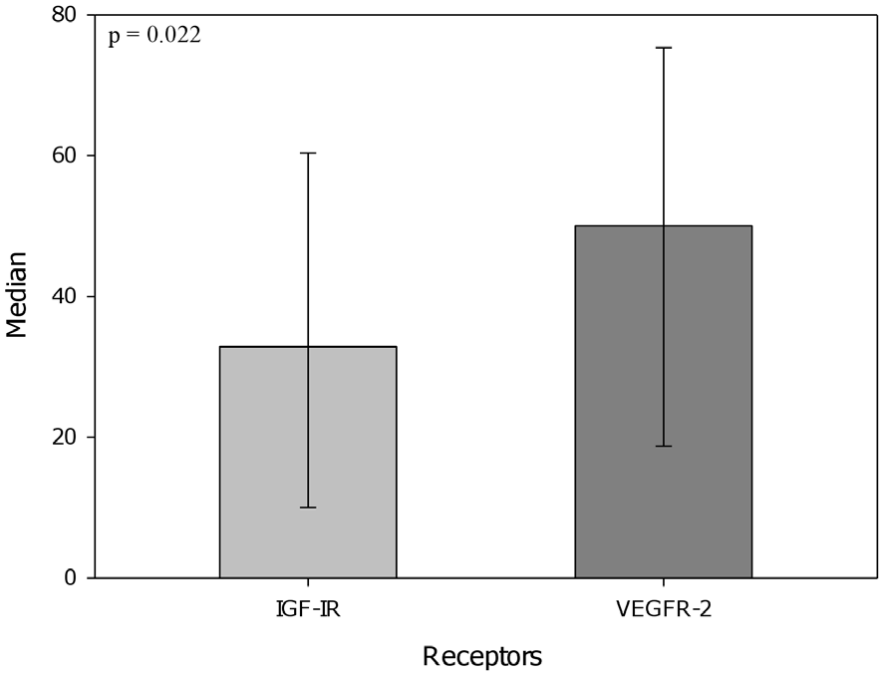
Median of percentage IGF-IR and VEGFR-2 expression on the CETCs in 50 breast cancer patients.

Using double staining experiments, IGF-IR-expressing EpCAM-positive CETCs were detected in 42 (84%) patients. The median percentage of CETCs expressing IGF-IR was 32.9% (range 0-83.3%). VEGFR-2 expressing EpCAM-positive CETCs were identified in 42 (84%) of patients. The percentage of CETCs expressing VEGFR-2 in addition to EpCAM ranged from 0% to 100% (median 50%). There was a statistically significant difference between the median of IGF-IR expression and VEGFR-2 expression on the CETCs (p<0.05) (Figure 5).

**Figure 6:**
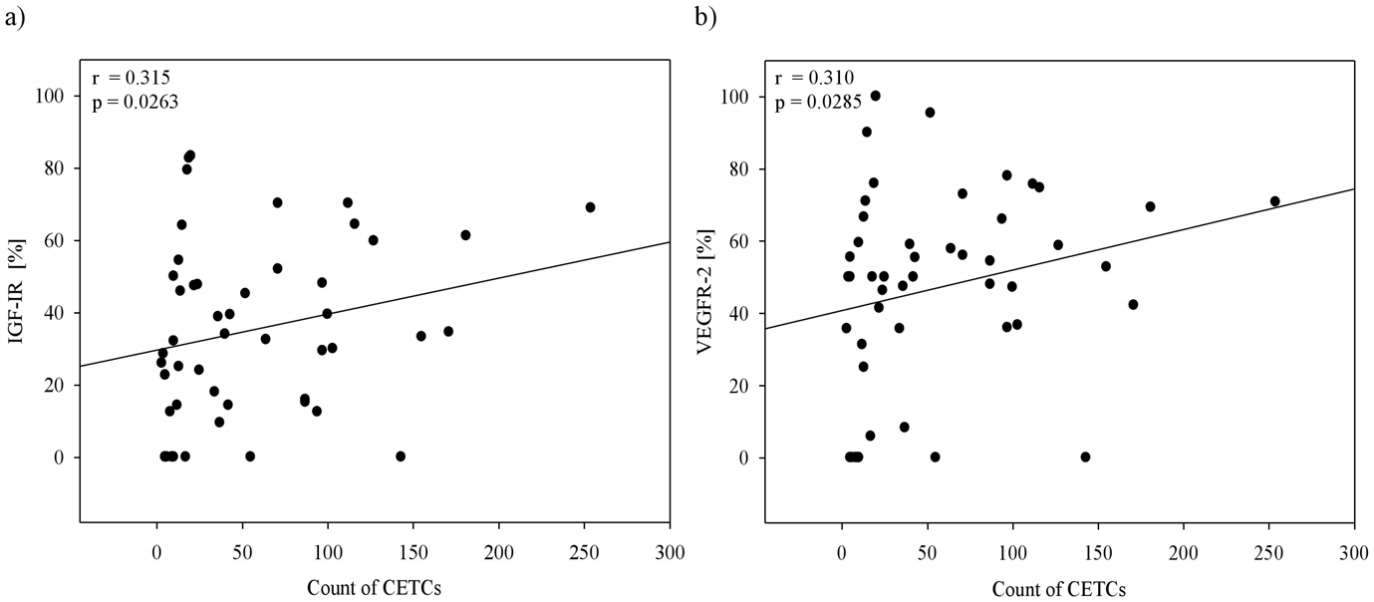
Correlation between the numbers of CETCs and the expression of receptors on the surface of CETCs in 50 breast cancer patients.

**Figure 7:**
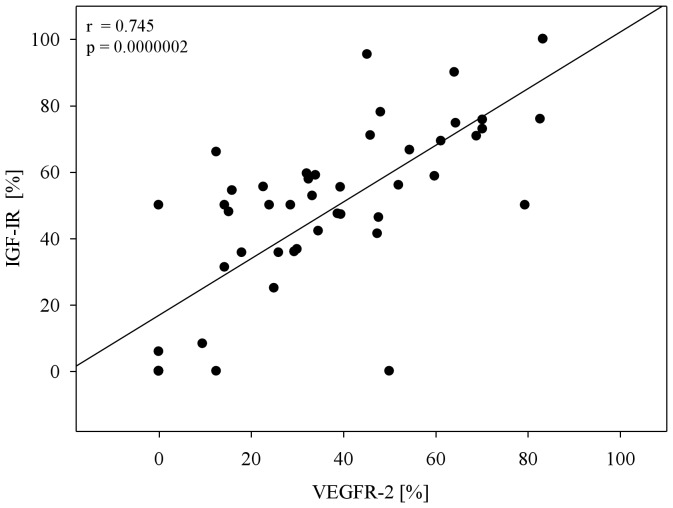
Correlation between the expression of IGF-IR and VEGFR-2 on the surface of CETCs in 50 breast cancer patients.

The correlation between the number of CETCs and the expression of IGF-IR (r = 0.315, p = 0.026) and VEGFR-2 (r = 0.0310, p = 0.028) was weak but statistically significant (Figure 6 a, b). However, according to the Spearman rank correlation analysis the expression of IGF-IR and VEGFR-2 was highly positively correlated and statistically highly significant (r = 0.745 and p = 0.0000002) (Figure 7). No relationship was found between IGF-IR, VEGFR-2 expression and tumor size, lymph node status, distant metastasis. The expression of either IGF-IR or VEGFR-2 on CETCs did not correlate with ER/PR and HER-2/neu status of the primary tumor. The co-expression of both receptors was confirmed in 40 patients (80%) and ranged between 70% and 100%.

## Discussion

It is well known that circulating epithelial tumor cells are a distinct population of cancer cells that have detached from the primary tumor and enter the blood circulation and can create a secondary tumor. The detection of tumor cells circulating in the peripheral blood of metastatic cancer patients has been associated with both disseminated disease and a higher risk of cancer progression [14]. The isolation of circulating tumor cells presents a tremendous technical challenge, because these cells are assumed to be rare, supposedly comprising a few cells per 10^6^ hematological cells in the blood of patients with cancer disease [15]. However, isolating other rare cells such as CD34^+^ stem cells is no longer a prerequisite for their enumeration and is rather considered counterproductive for the correct determination of the number of CD34-positive cells during stem cell mobilization [16]. The same pertains to the enumeration of the rare circulating epithelial antigen-positive cells, putative tumor cells in tumor-bearing patients. Therefore, we have developed the maintrac*®* method, an approach designed to minimize cell loss during the labeling and analysis process. Using this approach the number of CETCs from the peripheral blood of patients with solid tumors detected is tenfold higher than those detected with other methods [17]. This is due to the omission of all enrichment steps during the preparation, which includes no isolation steps apart from one centrifugation step after red blood cell lysis. A comparable approach, the CTC chip also omits all pre-analytical steps to minimize cell loss but uses adhesion to antibody-coated poles for epithelial antigen-positive cell enrichment, thereby resulting in the detection of higher numbers of tumor cells [18]. By contrast, the fixation and magnetic enrichment in the CellSearch*®* method leads not only to a heavy loss but also to a massive destruction of the tumor cells [17]. Furthermore, preservatives present in the CellSave tubes are known to provide good morphological preservation but poor antigen preservation due to the cross-linking mechanism of fixation [19]. In RT-PCR methods a considerable number of circulating tumor cells may be lost due to pre-analytical enrichment steps [15]. Thus, the maintrac*®* method, which is relatively nondissipative, is therefore very sensitive and very effective and, for this reason, more CETCs can be detected in comparison with other methods [13] [20].

Attempting to improve the understanding of molecular events and critical pathways involved in breast cancer and to develop targeted therapies requires the identification of novel targets that have high specificity for the molecules involved in cell growth, survival, migration, invasion, metastasis, apoptosis, cell-cycle progression and angiogenesis [21]. Biomarker analysis is important for pharmacological intervention. Several antagonists of the IGF and VEGF systems involved in the aggressiveness of breast cancer have been developed, some of which have entered clinical trials [1]. IGF-IR plays a major role in cancer cell proliferation and survival, and confers resistance to cytotoxic, hormonal and targeted therapies in breast cancer [21]. There is evidence that IGF-IR is overexpressed in cancer cells compared with normal tissues. Shimizu C et al [22] reported that IGF-IR was overexpressed in 43.8% of breast tumors, whereas Railo MJ et al [23] have shown a positive IGF-IR expression in 39% of breast cancer samples. Our current study demonstrates that the IGF-IR is also frequently expressed on the CETCs of patients with breast cancer, independent of the stage of the disease. In accordance with de Bono et al [24], we showed that 84% of patients additionally express IGF-IR on CETCs. We also postulate a relationship between IGF-IR expression and a more aggressive disease, because patients with high CETCs counts usually have high IGF-IR expression on these cells (r = 0.315, p = 0.0263). This suggests that IGF-IR may play an important role in the aggressiveness of circulating tumor cells and their ability to grow after adhesion and to form metastases. Furthermore, the signaling through the insulin-like growth factor I receptor has been implicated in the resistance to anti-cancer agents, including inhibitors of the HER family of receptors [25]. Two IGF-IR inhibitors are being evaluated in combination with existing HER2-directed therapies for the treatment of HER2-positive metastatic breast cancer in clinical studies because co-inhibition of the IGF and HER2 pathways may improve the efficiency of targeting these pathways [26]. The detection of IGF-IR-positive CETCs could be a predictive marker for patients who could benefit from anti-IGF-IR therapy, especially in Her-2-positive patients.

Angiogenesis is a fundamental process in tumor growth, dissemination, invasion and metastasis. Vascular endothelial growth factor (VEGF) and its receptor (VEGFR) play a pivotal role in both physiological and pathological angiogenesis. VEGFR-2 mediates the majority of VEGF-induced angiogenic effects. In invasive breast carcinomas Nakopoulou et al [27] detected VEGFR-2 in 64.5% of cases but VEGFR-2 expression did not correlate with the stage of the disease or patients’ survival. Kallergi G et al [11] showed that expression of VEGFR-2 on circulating tumor cells occurred in 70% of circulating tumor cells, but the expression of this receptor did not correlate with the ER/PR or HER-2/neu status of the primary tumor, and these results are consistent with our results. There is strong evidence that antiangiogenic therapy using VEGF/VEGFR inhibitors can result in a clinical benefit for cancer patients [28] and this may not only be due to inhibition of vessel formation but also to direct action on VEGFR-2-positive cancer cells. Therefore, using CETCs as a liquid biopsy could aid in selecting appropriate patients for targeted and personalized treatment strategies against cancer.

We found a weak but statistically significant correlation between the number of CETCs and the expression of IGF-IR or VEGFR-2; therefore, it can be assumed that IGF-IR or VEGFR-2 are frequently expressed in patients with higher numbers of CETCs. Additionally, the expression of these biomarkers could be relevant for the patient’s individual disease and treatment.

Furthermore, we found a significant linear correlation between IGF-IR expression and the presence of VEGFR-2 on the CETCs. Most of the CETCs stained for IGF-IR were also positive for VEGFR-2 expression. Therefore, IGF-IR and VEGFR-2 may represent important components of growth factor signaling in breast cancer. Even if there were no relationship between the expression of IGF-IR or VEGFR-2 on the CETCs and the stage of the disease, or the ER/PR or HER-2/neu status of the primary tumor, these receptors may be important for activating previously dormant cells due to e.g. inflammatory stimuli in order to grow into metastasis. Our results also confirmed the heterogeneity of CETCs, with the receptors examined in the present study expressed only in part of the examined CETCs of the same patient. This may be an explanation of diversity for the metastatic potential of CETCs. Our results demonstrated the expression of IGF-IR and VEGFR-2 on the CETCs in patients with breast cancer and thus contribute a basis for using anti-IGF-IR and anti-angiogenic therapy for their elimination.
